# Stigmatizing attitudes toward mental illness among mental health professionals in Kazakhstan: A cross-sectional study – ERRATUM

**DOI:** 10.1017/gmh.2026.10258

**Published:** 2026-07-13

**Authors:** Alina Abdusalyamova, Yerbol Nurkatov, Nabiy Yessimov, Raushan Alibekova

The publisher apologises for errors in the Kazakh language version of the abstract.

The abstract was published as follows:

**түйіндеме**

Б л зерттеуді ма саты Орталы Азияда ы е ірі табысы ортадан жо ары ел - аза станда ы психикалы денсаулы са тау мамандарыны психикалы ауруы бар адамдар а деген стигматизациялаушы к з арастарасымен байланысты факторларды ба алау болып табылады. 15 тарма тан т ратын Денсаулы са тау мамандарына арнал ан “Денсаулы Са тау Мамандары шін Ой Ашу Стигма Шкаласын” олдана отырып, аза станны барлы айма тарынан келген психиатрлар, наркологтар, психотерапевтер, психиатриялы резиденттер ж не психикалы денсаулы са тау мейірбикелері арасында к лдене онлайн сауалнама ж ргізілді. Стигманы орташа пайы 75-тен 40.1 болды, оны ішінде егде жаста ы мамандар (β = 3.32; 95% CI: 1.24, 5.39) ж не есірткіге т уелділік саласында ж мыс істейтін мамандарды арасында (β = 1.99; 95% CI: 0.32, 3.66) стигманы жо ары де гейі бай алды. Ер мамандарды (β = −3.29; 95% CI: −5.57, −1.02) стигма пайлары т мен болды. Психикалы денсаулы са тау ж мысын онша рметтелмейтін маманды ретінде абылдау (β = 6.43; 95% CI: 3.26, 9.60) ж не осы саланы о серіне к м ндану (β = 2.69, 95% CI: 0.52, 4.86) стигманы жо ары де гейімен байланысты болды; ал психикалы б зылулары бар туыстарыны немесе достарыны болуы (β = −2.36; 95% CI: −3.95, −0.77) ж не психотерапиямен б рын ы т жірибесі болуы (β = −2.12; 95% CI: −3.98, −0.26) стигманы т мен де гейімен байланысты болды. Б л т жырымдар денсаулы са тау мекемелеріндегі стигманы азайту ж не пациенттер мен медицина ызметкерлері шін олдаушы ж не инклюзивті орта жасау шін болаша та ма сатты араласулар а негіз бола алады. Т йін с здер: психикалы денсаулы , стигма, к з арас, психикалы денсаулы са тау мамандары, аза стан.

The correct abstract is as follows:

**Түйіндеме**

Бұл зерттеудің мақсаты Орталық Азиядағы ең ірі табысы ортадан жоғары ел - Қазақстандағы психикалық денсаулық сақтау мамандарының психикалық ауруы бар адамдарға деген стигматизациялаушы көзқарастарасымен байланысты факторларды бағалау болып табылады. 15 тармақтан тұратын Денсаулық сақтау мамандарына арналған “Денсаулық Сақтау Мамандары үшін Ой Ашу Стигма Шкаласын” қолдана отырып, Қазақстанның барлық аймақтарынан келген психиатрлар, наркологтар, психотерапевтер, психиатриялық резиденттер және психикалық денсаулық сақтау мейірбикелері арасында көлденең онлайн сауалнама жүргізілді. Стигманың орташа ұпайы 75-тен 40.1 болды, оның ішінде егде жастағы мамандар (β = 3.32; 95% CI: 1.24, 5.39) және есірткіге тәуелділік саласында жұмыс істейтін мамандардың арасында (β = 1.99; 95% CI: 0.32, 3.66) стигманың жоғары деңгейі байқалды. Ер мамандардың (β = −3.29; 95% CI: −5.57, −1.02) стигма ұпайлары төмен болды. Психикалық денсаулық сақтау жұмысын онша құрметтелмейтін мамандық ретінде қабылдау (β = 6.43; 95% CI: 3.26, 9.60) және осы саланың оң әсеріне күмәндану (β = 2.69, 95% CI: 0.52, 4.86) стигманың жоғары деңгейімен байланысты болды; ал психикалық бұзылулары бар туыстарының немесе достарының болуы (β = −2.36; 95% CI: −3.95, −0.77) және психотерапиямен бұрынғы тәжірибесі болуы (β = −2.12; 95% CI: −3.98, −0.26) стигманың төмен деңгейімен байланысты болды. Бұл тұжырымдар денсаулық сақтау мекемелеріндегі стигманы азайту және пациенттер мен медицина қызметкерлері үшін қолдаушы және инклюзивті орта жасау үшін болашақта мақсатты араласуларға негіз бола алады.

The Kazakh abstract has been updated in the article.

## References

[r1] Abdusalyamova A, Nurkatov Y, Yessimov N and Alibekova R. (2026) Stigmatizing attitudes toward mental illness among mental health professionals in Kazakhstan: A cross-sectional study. Cambridge Prisms: Global Mental Health, 13, e106. 10.1017/gmh.2026.10211 42246291 PMC13231235

