# Supplementary material for: Stigmatizing attitudes toward mental illness among mental health professionals in Kazakhstan: A cross-sectional study
Source: Glob Ment Health (Camb). 2026 Apr 30;13:e106. doi: 10.1017/gmh.2026.10211 (PMC13231235; doi:10.1017/gmh.2026.10211)
Supplement: Abdusalyamova et al. supplementary material [file S2054425126102118sup001.docx]

**Opening Minds Stigma Scale for Health Care Providers (OMS-HC) Kazakh version**

**“Денсаулық Сақтау Мамандары үшін Ой Ашу Стигма Шкаласы”**

Төмендегі мәлімдемелердің әрқайсысын оқып шығыңыз және оның психикалық ауруы бар адамдарға деген сезімдеріңізді қаншалықты сипаттайтынын бағалаңыз.

Төмендегі жауап нұсқаларының бірін таңдаңыз.

|  | Мәлімдеме | Толығымен келісемін | Келісемін | Жауап беруге қиналамын | Келіспеймін | Мүлдем келіспеймін |
| --- | --- | --- | --- | --- | --- | --- |
| 1 | Мен үшін психикалық ауруы бар адамға көмектескеннен гөрі физикалық ауруы бар адамға көмек көрсеткен ыңғайлы. |  |  |  |  |  |
| 2 | Егер әріптесім оның басқылаудығы немесе емделген психикалық ауруы бар екенін айтса, мен онымен бірге жұмыс істеуді жалғастыруға дайынмын. |  |  |  |  |  |
| 3 | Егер мен психикалық аурудан емделіп жүрген болсам, оны барлық әріптестерімнен жасырар едім. |  |  |  |  |  |
| 4 | Егер менде бар психикалық аурумен өз бетімше күресе алмасам, өзімді әлсіз сезінер едім. |  |  |  |  |  |
| 5 | Егер менде психикалық ауру болса, мен көмек сұрауға ұялушы едім. |  |  |  |  |  |
| 6 | Егер бақылаудағы немесе емделген психикалық ауруы бар қызметкер лауазымға ең қолайлы үміткер болса, жұмыс берушілер оны жұмысқа алуы керек. |  |  |  |  |  |
| 7 | Егер мен дәрігердің психикалық аурудан емделгенін білсем, мен әлі де сол дәрігерге барар едім. |  |  |  |  |  |
| 8 | Егер менде психикалық ауру болса, мен бұл туралы достарыма айтатын едім. |  |  |  |  |  |
| 9 | Менің кәсіби сенімдеріме қарамастан, психикалық аурулары бар адамдарға деген көзқарасым теріс. |  |  |  |  |  |
| 10 | Психикалық ауруы бар адамдарға көмектесу үшін қолымнан келетін нәрсе аз. |  |  |  |  |  |
| 11 | Психикалық аурулары бар адамдардың жартысынан көбі жақсару үшін жеткілікті түрде тырыспайды. |  |  |  |  |  |
| 12 | Мен психикалық ауруы бар адамның балалармен жұмыс істеуін қаламас едім, тіпті оның ауруы дұрыс бақыланса немесе емделсе де. |  |  |  |  |  |
| 13 | Медицина мамандары психикалық ауруы бар адамдарды қорғауға міндетті емес. |  |  |  |  |  |
| 14 | Психикалық дерті бар адам көрші тұрса, қарсы болмас едім. |  |  |  |  |  |
| 15 | Мен үшін психикалық ауруы бар адамға жанашырлық таныту қиын. |  |  |  |  |  |
